# Risk factors analysis of cognitive frailty among geriatric adults in nursing homes based on logistic regression and decision tree modeling

**DOI:** 10.3389/fnagi.2024.1485153

**Published:** 2024-11-21

**Authors:** Jing Gao, Dingxi Bai, Huan Chen, Xinyu Chen, Huan Luo, Wenting Ji, Chaoming Hou

**Affiliations:** College of Nursing, Chengdu University of Traditional Chinese Medicine, Chengdu, China

**Keywords:** cognitive frailty, risk factors, logistic regression, decision tree, nursing homes

## Abstract

**Objective:**

To investigate the risk factors associated with cognitive frailty among older adults in nursing homes using logistic regression and decision tree modeling, and to compare the predictive performance of these methods.

**Methods:**

A cross-sectional study was conducted involving 697 participants aged 60 and older residing in eight nursing homes in Sichuan province, China. Participants were recruited using convenience sampling. Data were collected through questionnaires administered to the older adults. Logistic regression and decision tree modeling were employed to construct models predicting cognitive frailty.

**Results:**

Logistic regression analysis identified age, education degree, exercise, intellectual activities, number of chronic diseases, nutritional status, sleep quality, and depression as significant predictors of cognitive frailty (all *p* < 0.05). The final decision tree model consisted of three layers and 17 nodes. Six factors were identified as significant predictors: sleep quality, number of chronic diseases, depression, education level, nutrition, and exercise. Receiver operating characteristic (ROC) curve analysis revealed that the area under the curve (AUC) for the logistic regression model was 0.735 (95% *CI*: 0.701–0.767) with a sensitivity of 0.58 and specificity of 0.75. The AUC for the decision tree model was 0.746 (95% *CI*: 0.712–0.778) with a sensitivity of 0.68 and specificity of 0.70.

**Conclusion:**

Age, education level, exercise, intellectual activities, sleep quality, number of chronic diseases, nutritional status, and depression are significant risk factors for cognitive frailty in older adults residing in nursing homes. Both logistic regression and decision tree models demonstrated comparable predictive performance, with each offering distinct advantages. The combined use of these methods can enhance predictive accuracy and provide valuable insights for clinical practice and policy development.

## Introduction

Frailty is a clinical condition characterized by an individual’s increased vulnerability to stressors due to the cumulative decline of multiple physiological systems associated with aging. This decline encompasses physical, cognitive, psychological, and social dimensions (Clegg et al., 2013; Kolle et al., 2023). According to the World Health Organization (WHO) Health Statistics Report 2021, approximately one in 10 of the global population is elderly (Li et al., 2022b). The growing aging population has become a significant public health concern. The increasing number of older adults poses substantial challenges for the global community, particularly regarding their health. Aging can lead to both physical frailty and cognitive decline (Li et al., 2022b). Recent studies have demonstrated a close association between physical frailty and cognitive decline, often occurring simultaneously during the aging process (Sugimoto et al., 2022). To comprehensively define this condition and expand the concept of multiple dimensions of frailty, the International Consensus Group from the International Academy of Nutrition and Aging (IANA) and the International Association of Gerontology and Geriatrics (IAGG) introduced the concept of cognitive frailty (CF). CF is a heterogeneous clinical manifestation that coexists with physical frailty and cognitive impairment but excludes Alzheimer’s disease and various types of dementia (Kelaiditi et al., 2013; Li et al., 2022b). In contrast to other forms of cognitive impairment, the international consensus group emphasized that CF is primarily caused by physical conditions rather than neurodegenerative diseases. Moreover, cognitive frailty may serve as a precursor to neurodegenerative processes (Sugimoto et al., 2022).

Since its introduction, the concept of cognitive frailty has garnered significant attention and has been extensively researched in a relatively short period. A systematic review of 51 studies conducted in 10 countries revealed that the overall prevalence of cognitive frailty among older adults worldwide was 16% (Zhang et al., 2021). Additionally, studies have shown that the incidence of cognitive frailty increases with age, rising from 4% in the 60–69 age group to 7% in the 70–79 group, then sharply to 29% for those 80 and older, culminating in a striking 50.3% for individuals aged 90 and above (Hao et al., 2018; Liu et al., 2023). Given the accelerating pace of aging and the increasing number of older adults, addressing cognitive frailty in geriatric populations is essential to prevent falls, disability, hospitalization, and other adverse outcomes (Huang J. et al., 2023).

Nursing homes, as primary care settings for older adults, play a crucial role in identifying, preventing, and managing cognitive frailty (Chen et al., 2023). Growing evidence suggests the importance of focusing on cognitive frailty among older individuals in nursing homes. For instance, a recent study (Huang J. H. et al., 2023) found a higher prevalence of cognitive frailty in nursing homes (49.07%) compared to communities (14.20%). Another study (Guo et al., 2023) conducted a meta-analysis and reported a prevalence of cognitive frailty among older adults in nursing homes of 24%, which exceeded that in the community (22%) and hospital (9%). When frail older individuals experience cognitive dysfunction combined with other health problems that impede their ability to manage daily activities, and family caregivers are unable to provide adequate support, placement in a nursing home may become necessary or even the only viable option (Seiger Cronfalk et al., 2017). However, older adults in nursing homes often face multiple health challenges, including advanced age, disability, dementia, and comorbid conditions (Gentili et al., 2022). Furthermore, the specialized management and living environment of nursing homes can limit physical activity, interpersonal communication, and emotional support with the outside world (Pastor-Barriuso et al., 2020). These complex factors contribute to poorer physical function, cognitive function, psychological well-being, and a higher risk of cognitive frailty among older adults in nursing homes.

Due to declining health, increased life expectancy, and decreased self-care abilities, an increasing number of older adults are opting to reside in nursing homes to receive professional care (Liu et al., 2022). The International Association of Gerontology and Geriatrics has emphasized the need for more research within nursing home settings (Liau et al., 2021), making cognitive frailty among older individuals in nursing homes a particularly worthy area of focus. While existing studies have primarily examined the prevalence and adverse outcomes of cognitive frailty, there is a growing emphasis on identifying modifiable risk factors. For instance, a meta-analysis explored modifiable risk factors of cognitive frailty in community-dwelling older adults (Yuan and Zhang, 2024). Besides, some studies have analyzed the risk factors of cognitive frailty in older patients with chronic diseases, such as diabetes (Deng et al., 2023), chronic kidney disease (Luo et al., 2022), chronic obstructive pulmonary disease (Wu et al., 2024), or cancer (Li et al., 2024). However, there remains a dearth of research focusing on the risk factors leading to cognitive frailty in older adults residing in nursing homes. Therefore, it is crucial to investigate the risk factors associated with cognitive frailty in this population to provide valuable insights and opportunities for early interventions to prevent cognitive frailty and mitigate the healthcare and financial burdens it imposes (Lee et al., 2023).

Understanding the factors that influence the development of CF among older adults in nursing homes is essential. Studies have demonstrated that logistic regression analysis models, in conjunction with decision tree models, can be valuable tools for analyzing risk factors and enhancing analytical efficacy (Rosenblatt and Yanez, 2022; Wang et al., 2024). This study aimed to investigate the risk factors associated with cognitive frailty in older adults residing in nursing homes using logistic regression analysis and decision tree models. The findings of this research can provide valuable insights for healthcare professionals to implement targeted interventions to prevent cognitive frailty in this population.

## Materials and methods

### Research design

A cross-sectional study was conducted from December 2021 to February 2022 in eight nursing homes located in Sichuan Province, China. Convenience sampling was employed to recruit participants. The sample size was determined based on the requirements for cross-sectional studies (Wan and Liu, 2007), resulting in a minimum sample size of 691 cases.

### Participants

Face-to-face investigations were conducted among older adults residing in nursing homes between December 2021 and February 2022. Participants were included if they were aged 60 or older, had resided in a nursing home for at least 3 months, exhibited clear consciousness and good language expression abilities, and volunteered to participate in the study. Exclusion criteria included severe visual impairment, hearing impairment, mental disorders, inability to complete physical function tests, Alzheimer’s disease or other types of dementia, and current participation in other clinical research trials.

### Ethical statement

This study adhered to the principles outlined in the Declaration of Helsinki. All participants provided informed consent, and the study was approved by the Jinniu District People’s Hospital Ethics Committee of Chengdu (No. QYYLL-2022-011).

### Research tools

#### General information questionnaire

Demographic information, life behavior habits, health conditions, and current medications were collected during the survey process. Demographic information included age, sex, education level, personal monthly income, marital status, frequency of family visits, pre-retirement occupation, and living environment. Life behavior habits encompassed smoking and drinking status, exercise frequency, and engagement in intellectual activities. Health conditions and current medications included the use of walking aids, self-rated health status, history of stress in the past year, type of medication, and the number of chronic diseases.

#### Physical frailty

The physical frailty status of participants was assessed using the Frailty Phenotype (FP) (Fried et al., 2001). This tool evaluates physical frailty based on five traits: weight loss, exhaustion, low physical activity, slow walking speed and low grip strength. Each trait was answered with “Yes/no.” A “yes” answer was worth 1 point, and a “no” answer was worth 0 point. The total score was 0–5 points. Participants were categorized as frail (≥3 points), pre-frail (1–2 points), or non-frail (0 points) according to their FP scores (Wu et al., 2022). The Cronbach’s *α* of the FP in this study was 0.858, indicating good internal consistency.

#### Cognitive function

The cognitive function of older adults was assessed using the Chinese version of the Mini-Mental State Examination (MMSE) (Wang et al., 2024). The MMSE comprises five dimensions: orientation, memory, attention and calculation, recall, and language ability, with a total of 30 items. Scores range from 0 to 30, with lower scores indicating poorer cognitive function. Individuals with an MMSE score of ≤21 (illiterate), ≤24 (primary school education), or ≤27 (secondary school education and above) are considered to have mild cognitive impairment. The Cronbach’s *α* of MMSE in this study was 0.779.

#### Cognitive frailty

According to the previous study (Alqahtani and Alenazi, 2023), CF was defined as a Frailty Phenotype (FP) score of ≥3 and an MMSE score of 18 to <24 in the absence of dementia.

#### Sleep quality

The sleep quality of participants was assessed using the Athens Insomnia Scale (AIS), a validated instrument with a Cronbach’s *α* of 0.83 and a test–retest reliability of 0.94 (Soldatos et al., 2003). The AIS consists of eight items with a total possible score ranging from 0 to 24. In our study, the Cronbach’s *α* of the AIS was found to be 0.858. The AIS scoring scale classifies sleep disorders as follows: a score below 4 indicates no sleep disorder, a score between 4 and 6 suggests suspected insomnia, and a score above 6 confirms a diagnosis of insomnia.

#### Nutritional status

The nutritional status of participants was assessed using the Mini Nutritional Assessment Short Form (MNA-SF), a six-item scale with a Cronbach’s alpha of 0.828 in this study. The MNA-SF evaluates eating behavior, weight change, activity level, recent illness or psychological trauma, mental and psychological well-being, and body mass index. A total score of 0–14 points is assigned, with 0–7 indicating malnutrition, 8–11 indicating risk of malnutrition, and 12–14 indicating normal nutrition (Rubenstein et al., 2001).

#### Depression

The Geriatric Depression Scale-15 (GDS-15) is a widely used instrument for assessing depressive symptoms in older adults. It demonstrates good reliability, with a Cronbach’s alpha of 0.793 and a test–retest reliability of 0.728 in the Chinese elderly population (Tang, 2013). In the present study, the GDS-15 exhibited a Cronbach’s alpha of 0.823, further confirming its reliability. This 15-item scale measures negative emotions experienced in the past week, with a total score ranging from 0 to 15 points. A score of 8 or higher indicates the presence of depressive symptoms, and a higher score signifies more severe depression.

#### Anxiety

The Generalized Anxiety Disorder 7 (GAD-7) scale, a seven-item tool used to assess anxiety symptoms in older adults, has a total score ranging from 0 to 21 points. A score of 5 or higher indicates the presence of anxiety symptoms, with higher scores reflecting more severe anxiety. The Chinese version of GAD-7 has demonstrated Cronbach’s *α* of 0.898 and test–retest reliability of 0.856 (He et al., 2010). In the current study, the GAD-7 scale exhibited a Cronbach’s *α* of 0.796.

#### Social support

The Social Support Rating Scale (SSRS), with a Cronbach’s *α* of 0.808 in our study, was used to measure the social support level of participants. The scale consists of 10 items with a total score of 66 points. Higher scores indicate higher levels of social support, with scores ≤22 classified as low, 23–44 as medium, and 45–66 as high. Previous research has established the reliability and validity of the Chinese version of the SSRS (Liu et al., 2008).

### Statistical analysis

To mitigate selection bias and control for potential confounders, we established clear inclusion and exclusion criteria to ensure sample representativeness. In the statistical analysis, cognitive frailty served as the dependent variable, while statistically significant independent variables (*p* < 0.05) identified in univariate analysis were included in the multivariate logistic regression and decision tree models to analyze factors influencing cognitive frailty in nursing home residents. The decision tree model was constructed using the Classification and Regression Tree (CART) algorithm with a minimum of 50 cases for the parent node and 20 cases for the child node. In addition, considering that the meaning of exercise and education degree in the independent variables overlapped with that of FP (the item of physical activities) and MMSE (the evaluation results depended on the education degree) in assessing cognitive frailty, respectively, we excluded exercise and education degree in the independent variables for sensitivity analysis and analyzed the stability of the results. Receiver operating characteristic (ROC) curves of both models were generated using MedCalc 20.1 software, with AUC, specificity, and sensitivity employed to evaluate their predictive performance. Statistical analyses were conducted using SPSS 23.0 software, incorporating descriptive statistics for general information (frequency counts, constitutive ratios, means, and standard deviations).

## Results and discussion

### Results

#### Description of each indicator

In this study, 720 questionnaires were distributed, resulting in 697 valid responses, yielding a recovery rate of 96.81%. Of the participants, 225 (32.28%) were classified as having cognitive frailty, while 472 (67.72%) were classified as not having cognitive frailty.

#### Comparison of the characteristics between CF and non-CF older people in nursing homes

Participants were categorized into two groups based on the presence or absence of CF: the CF group (*n* = 225) and the non-CF group (*n* = 472). This classification revealed a prevalence of CF in nursing homes at 32.28%. A comparison of general information, physical functions, psychological health, and social support between the two groups revealed statistically significant differences in age, education level, personal monthly income, pre-retirement occupation, smoking history, exercise, intellectual activities, use of walking aids, self-assessed health status, type of medication, number of chronic diseases, nutritional status, sleep quality, depression, and social support (*p* < 0.05), as presented in Table 1. No significant differences were observed in terms of sex, marital status, dwelling environment, frequency of family visits, drinking history, history of stress in the past year, and anxiety (*p* > 0.05).

**Table 1:** Comparison of the occurrence of CF among older adults in nursing homes with different characteristics.

Variable		Cognitive frailty [*n* = 225(%)]	Non-cognitive frailty [*n* = 472(%)]	Prevalence (totally = 32.28%)	*χ^2^/Z*	*P*
Age	60 ~ 69	20 (8.89%)	77 (16.31%)	20.62%	−6.260^b^	<0.001
	70 ~ 79	44 (19.56%)	123 (26.06%)	26.35%		
	80 ~ 89	79 (35.11%)	219 (46.40%)	26.51%		
	≥90	82 (36.44%)	53 (11.23%)	60.74%		
Sex	Men	104 (46.22%)	221 (46.82%)	32.00%	0.022^a^	0.882
	Women	121 (53.78%)	251 (53.18%)	32.53%		
Education degree	Illiteracy	71 (31.56%)	57 (12.08%)	55.47%	−6.264^b^	<0.001
	Secondary schools	68 (30.22%)	133 (28.18%)	33.83%		
	Middle school (Junior/High/Secondary)	64 (28.44%)	194 (41.10%)	24.81%		
	College and above	22 (9.78%)	88 (18.64%)	20.00%		
Marital status	Married with spouse	51 (22.67%)	125 (26.48%)	28.98%	1.176^a^	0.278
	Unmarried/divorced/widowed	174 (77.33%)	347 (73.52%)	33.40%		
Monthly personal income	<1,000	64 (28.44%)	30 (6.36%)	68.09%	−5.795^b^	<0.001
	1,000 ~ 2,999	73 (32.44%)	178 (37.71%)	29.08%		
	3,000 ~ 4,999	61 (27.11%)	179 (37.92%)	25.42%		
	≥5,000	27 (12.00%)	85 (18.01%)	24.11%		
Living environment	Single room	43 (19.11%)	65 (13.77%)	39.81%	3.762^a^	0.152
	Double room	125 (55.56%)	268 (56.78%)	31.81%		
	Multi-room apartment	57 (25.33%)	139 (29.45%)	29.08%		
Pre-retirement occupation	Mental labor is the mainstay	71 (31.56%)	188 (39.83%)	27.41%	4.468^a^	0.035
	Physical labor is the mainstay	154 (68.44%)	284 (60.17%)	35.16%		
Frequency of family visits (times/month)	0	5 (2.22%)	4 (0.85%)	55.56%	−1.389^b^	0.165
	<1	81 (36.00%)	147 (31.14%)	35.53%		
	1 ~ 3	98 (43.56%)	231 (48.94%)	29.79%		
	≥4	41 (18.22%)	90 (19.07%)	31.30%		
Smoking history	Yes	104 (46.22%)	167 (35.38%)	38.38%	7.535^a^	0.006
	No	121 (53.78%)	305 (64.62%)	28.40%		
Drinking history	Yes	86 (38.22%)	149 (31.57%)	36.60%	3.019^a^	0.082
	No	139 (61.78%)	323 (68.43%)	30.09%		
Exercise	Never	44 (19.56%)	27 (5.72%)	61.97%	38.516^a^	<0.001
	Occasionally	98 (43.56%)	189 (40.04%)	34.15%		
	Frequently	83 (36.89%)	256 (54.24%)	24.48%		
Intellectual activities	Never	43 (19.11%)	52 (11.02%)	45.26%	8.976^a^	0.011
	Occasionally	108 (48.00%)	236 (50.00%)	31.40%		
	Frequently	74 (32.89%)	184 (38.98%)	28.68%		
Use of walking aids	Yes	118 (52.44%)	208 (44.07%)	36.20%	4.294^a^	0.038
	No	107 (47.56%)	264 (55.93%)	28.84%		
Self-assessed health status	Well	46 (20.44%)	142 (30.08%)	24.47%	18.444^a^	<0.001
	General	87 (38.67%)	210 (44.49%)	29.29%		
	Bad	92 (40.89%)	120 (25.42%)	43.40%		
History of stress in the past 1 year	Yes	80 (35.56%)	201 (42.58%)	28.47%	3.129^a^	0.077
	No	145 (64.44%)	271 (57.42%)	34.86%		
Type of medication	0	29 (12.89%)	77 (16.31%)	27.36%	−2.320^b^	0.020
	1 ~ 2	66 (29.33%)	153 (32.42%)	30.14%		
	3 ~ 4	72 (32.00%)	161 (34.11%)	30.90%		
	≥5	58 (25.78%)	81 (17.16%)	41.73%		
Number of chronic diseases	0	21 (9.33%)	78 (16.53%)	21.21%	−3.640^b^	<0.001
	1 ~ 2	61 (27.11%)	139 (29.45%)	30.50%		
	3 ~ 4	69 (30.67%)	161 (34.11%)	30.00%		
	≥5	74 (32.89%)	94 (19.92%)	44.05%		
Nutrition status	Normal nutrition	43 (19.11%)	133 (28.18%)	24.43%	−3.895^b^	<0.001
	Risk of malnutrition	90 (40.00%)	214 (45.34%)	29.61%		
	Malnutrition	92 (40.89%)	125 (26.48%)	42.40%		
Sleep quality	No sleep disorders	45 (20.00%)	154 (32.63%)	22.61%	−4.077^b^	<0.001
	Suspected insomnia	87 (38.67%)	185 (39.19%)	31.99%		
	Insomnia	93 (41.33%)	133 (28.18%)	41.15%		
Depression	Yes	79 (35.11%)	117 (24.79%)	40.31%	8.033^a^	0.005
	No	146 (64.89%)	355 (75.21%)	29.14%		
Anxiety	Yes	49 (21.78%)	94 (19.92%)	34.27%	0.324^a^	0.569
	No	176 (78.22%)	378 (80.08%)	31.77%		
Social support	Low level	78 (34.67%)	103 (21.82%)	43.09%	−3.228^b^	0.001
	Mid-level	101 (44.89%)	246 (52.12%)	29.11%		
	High level	46 (20.44%)	123 (26.06%)	27.22%		

^aχ2 test; bMann–Whitney U test.^

##### Multivariate logistic regression analysis

When cognitive frailty was used as the dependent variable (no = 0, yes = 1), 15 factors were found to be statistically significant in univariate analysis and were subsequently included as independent variables in the multivariate logistic regression model: age, education degree, personal monthly income, pre-retirement occupation, smoking history, exercise, intellectual activities, use of walking aids, self-assessment of health status, type of medication, number of chronic diseases, nutritional status, sleep quality, depression, and social support. Multivariate logistic regression revealed that age, education degree, exercise, intellectual activities, number of chronic diseases, nutritional status, sleep quality, and depression were significant risk factors for cognitive frailty among older adults in nursing homes (*p* < 0.05), as shown in Table 2.

**Table 2:** Multivariate logistic regression analysis of factors influencing cognitive frailty in elderly people in nursing homes.

Variables	*β*	*SE*	Wald value	*p-*value	OR value	95% CI
Age (years old) (reference: 60 ~ 69)						
70 ~ 79	0.380	0.364	1.088	0.297	1.462	0.716 ~ 2.984
80 ~ 89	0.798	0.316	6.380	0.012	2.222	1.196 ~ 4.129
≥90	1.331	0.351	14.392	<0.001	3.786	1.903 ~ 7.532
Education degree (reference: illiteracy)						
Secondary schools	−0.203	0.267	0.579	0.447	0.816	0.483 ~ 1.377
Middle school	−0.597	0.260	5.267	0.022	0.551	0.331 ~ 0.917
College and above	−1.106	0.333	11.027	0.001	0.331	0.172 ~ 0.636
Exercise (reference: never)						
Occasionally	−0.366	0.307	1.421	0.233	0.694	0.380 ~ 1.266
Frequently	−0.671	0.305	4.842	0.028	0.511	0.281 ~ 0.929
Intellectual activities (reference: never)						
Occasionally	−0.506	0.273	3.449	0.063	0.603	0.353 ~ 1.028
Frequently	−0.617	0.281	4.839	0.028	0.539	0.311 ~ 0.935
Number of chronic diseases (reference: 0)						
1 ~ 2	0.380	0.331	1.319	0.251	1.462	0.765 ~ 2.797
3 ~ 4	0.701	0.322	4.739	0.029	2.015	1.072 ~ 3.786
≥5	1.180	0.344	11.79	0.001	3.253	1.659 ~ 6.379
Nutrition status (reference: normal nutrition)						
Risk of malnutrition	0.602	0.264	5.196	0.023	1.826	1.088 ~ 3.064
Malnutrition	0.838	0.261	10.333	0.001	2.312	1.387 ~ 3.853
Sleep quality (reference: no sleep disturbance)						
Suspected insomnia	0.732	0.249	8.663	0.003	2.078	1.277 ~ 3.383
Insomnia	1.334	0.289	21.35	<0.001	3.795	2.155 ~ 6.682
Depression (reference: no depression)						
No	0.799	0.224	12.678	<0.001	2.223	1.432 ~ 3.450

##### Decision tree modeling analysis of factors influencing cognitive frailty in nursing-homes older adults

**Figure 1:**
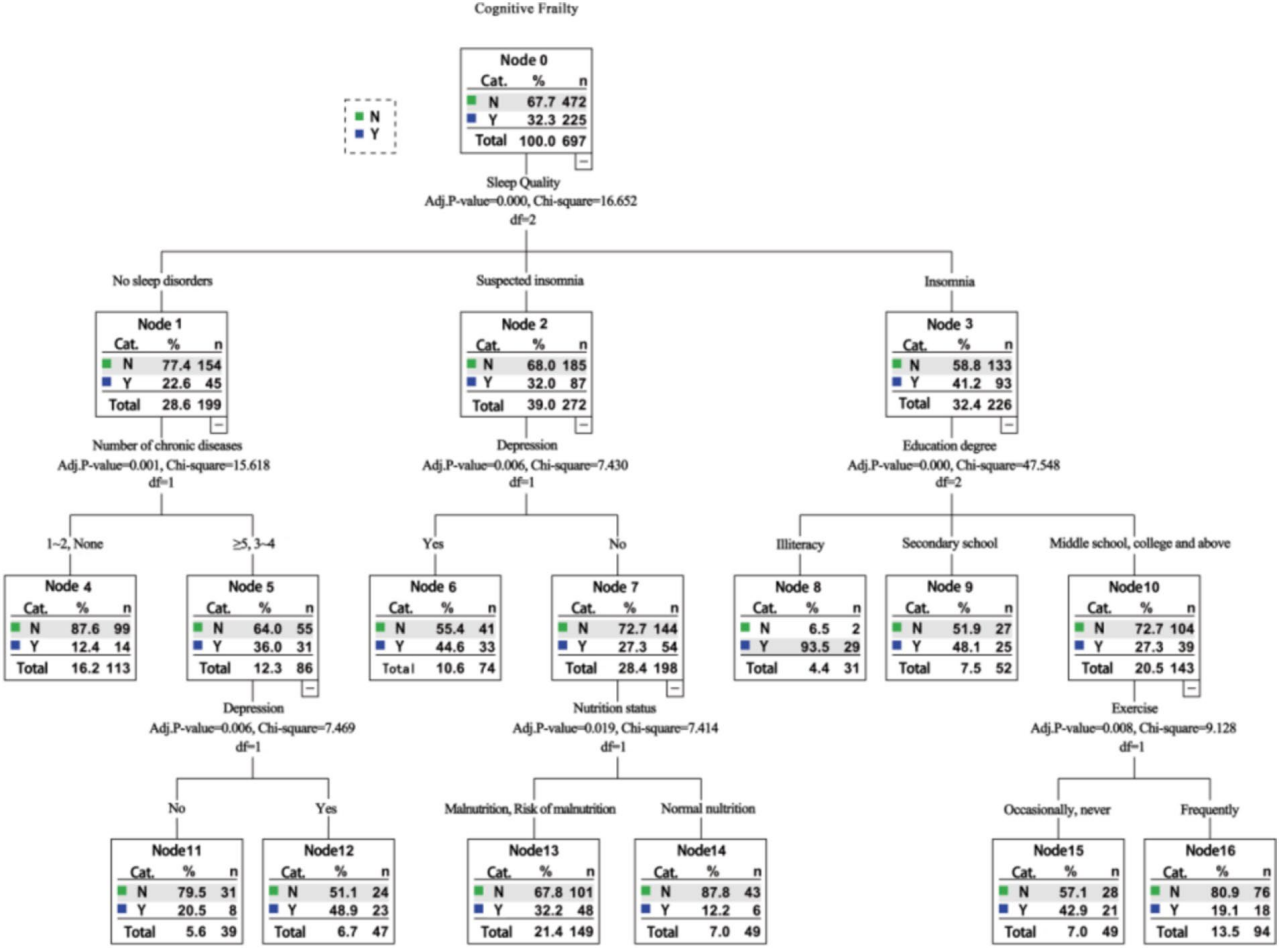
Decision tree model analysis of factors influencing cognitive frailty among old adults in nursing homes.

The Chi-squared Automatic Interaction Detection (CHAID) algorithm was employed to construct a decision tree, with a significance level of 0.05 for branch splitting. The minimum sample size for parent and child nodes was set at 50 and 20, respectively. The variables included in the decision tree analysis were consistent with those used in the multivariate logistic regression analysis. As illustrated in Figure 1, the resulting decision tree comprised three levels, one terminal node, 10 child nodes, and identified six variables associated with cognitive frailty among older adults in nursing homes: sleep quality, number of chronic diseases, depression, education level, nutritional status, and exercise.

##### Sensitivity analysis

**Figure 2:**
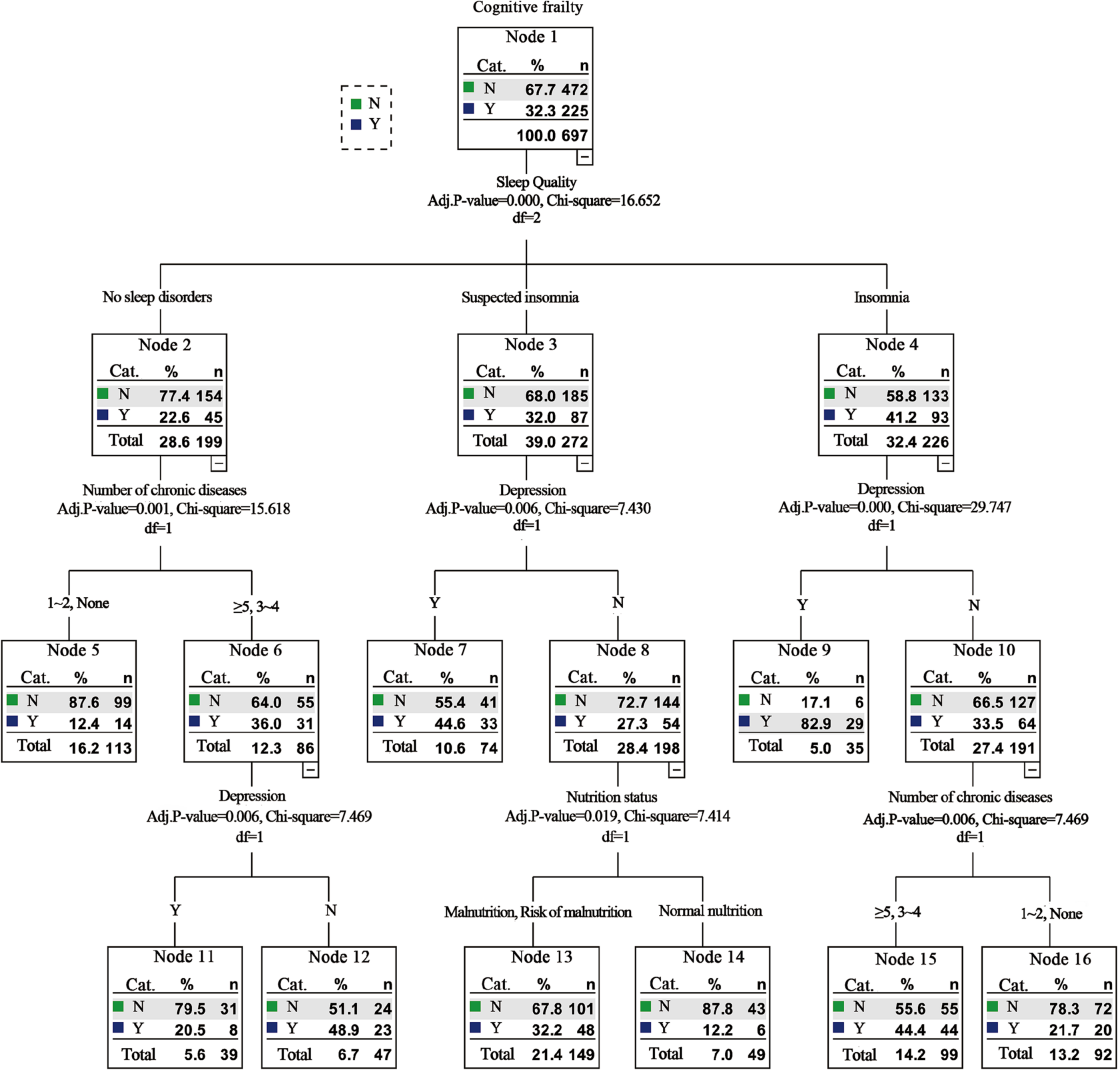
Sensitivity analysis results of decision tree model analysis of factors influencing cognitive frailty among old adults in nursing homes.

After we deleted the exercise and education degree to conduct the sensitivity analysis, the results of logistic regression analysis showed that the remaining factors of age, intellectual activities, number of chronic diseases, nutrition status, sleep quality, and depression still the risk factors of CF among the older adults in nursing homes. And results of the decision tree model analysis indicated that the sleep quality, number of chronic diseases, depression, and nutritional status were also still associated with CF in nursing homes. Besides, it suggested that sleep had the most significant effect on CF and was the most important influencing factor, which was consistent with previous results (Table 3 and Figure 2).

**Table 3:** Sensitivity analysis results of multivariate logistic regression analysis of factors influencing cognitive frailty in elderly people in nursing homes.

Variables	*β*	*SE*	*P-*value	95% CI
Age (reference: 60 ~ 69)				
70 ~ 79	0.209	0.351	0.552	0.619 ~ 2.454
80 ~ 89	0.795	0.309	0.010	1.208 ~ 4.058
≥90	1.370	0.343	<0.001	2.007 ~ 7.710
Intellectual activities (reference: never)				
Occasionally	−0.598	0.268	0.026	0.325 ~ 0.931
Frequently	−0.721	0.276	0.009	0.283 ~ 0.835
Number of chronic diseases (reference: 0)				
1 ~ 2	0.379	0.322	0.239	0.777 ~ 2.749
3 ~ 4	0.687	0.312	0.028	1.078 ~ 3.669
≥5	1.122	0.334	0.001	1.596 ~ 5.908
Nutrition status (reference: normal nutrition)				
Risk of malnutrition	0.624	0.258	0.016	1.125 ~ 3.095
Malnutrition	0.882	0.255	0.001	1.466 ~ 3.983
Sleep quality (reference: no sleep disturbance)				
Suspected insomnia	0.722	0.243	0.003	1.278 ~ 3.315
Insomnia	1.174	0.278	<0.001	1.877 ~ 5.575
Depression (reference: no depression)				
Yes	0.887	0.218	<0.001	1.582 ~ 3.724

##### Comparison of the predictive efficiency of the decision tree model and logistic regression model

**Figure 3:**
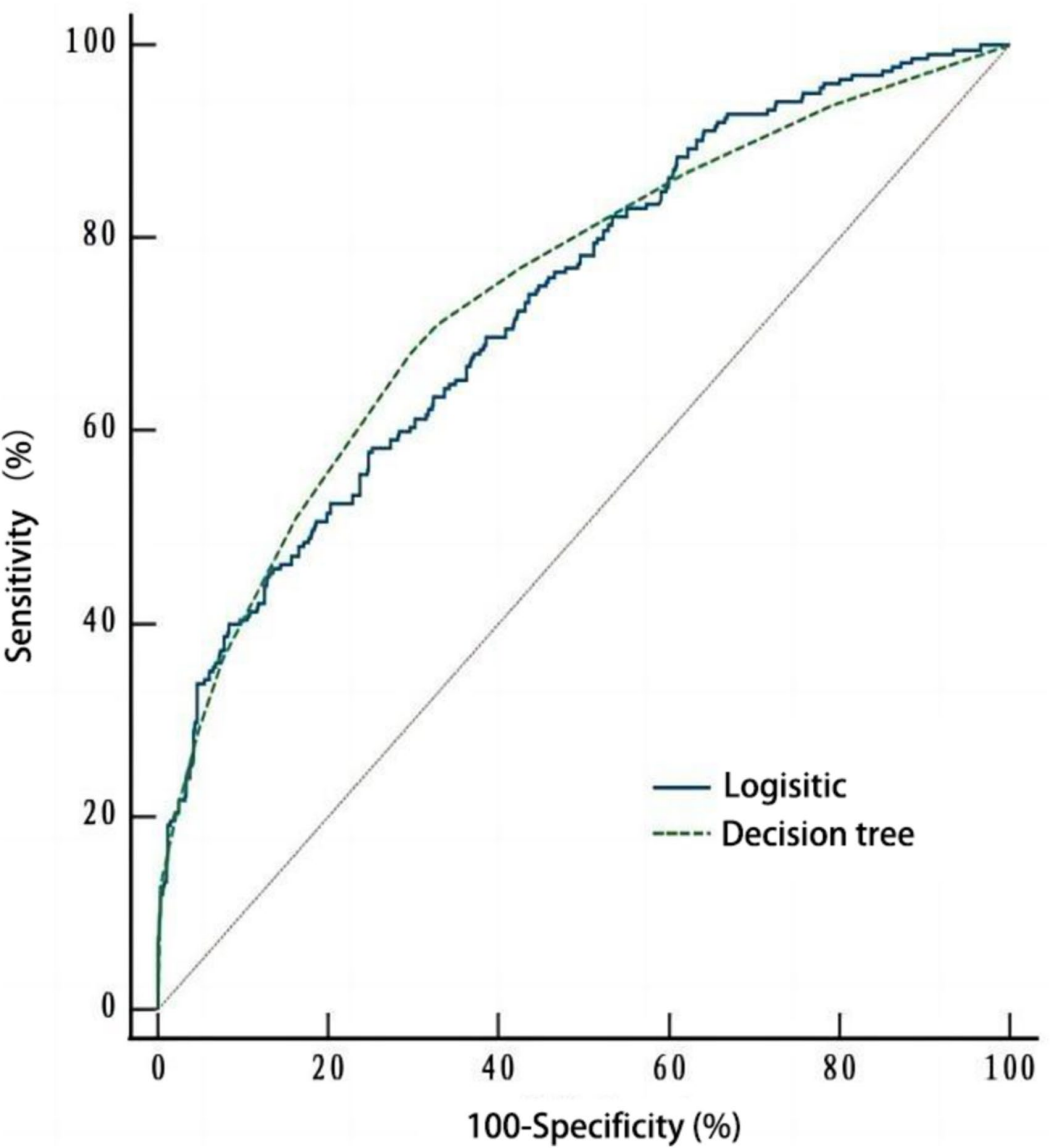
The ROC curves of the logistic regression model and decision tree model.

As depicted in Figure 3, ROC curves were plotted based on the influencing factor models constructed using logistic regression and decision tree modeling. The area under the ROC curve (AUC) for the logistic regression model was 0.735 (95% *CI*: 0.701–0.767, *p* < 0.001), with a sensitivity of 0.582 and a specificity of 0.748. The AUC for the decision tree model was 0.746 (95% *CI*: 0.712–0.778, *p* < 0.001), with a sensitivity of 0.680 and a specificity of 0.703. Moreover, no significant difference (*Z* = 0.465, *p* = 0.642) was observed in the AUC between the two models (Table 4).

**Table 4:** Comparison of classification effects of the logistic regression and decision tree models.

Model	*AUC*	*SE*	95% CI	*P*	Specificity	Sensitivity
Logistic regression	0.735	0.020	0.701 ~ 0.767	<0.001	0.748	0.582
Decision tree	0.746	0.020	0.712 ~ 0.778	<0.001	0.703	0.680

### Discussion

This study initially analyzed the influencing factors of cognitive frailty among older adults in nursing homes. Unlike previous research (Liu et al., 2022; Zhang et al., 2024), our analysis encompassed a broader range of factors using both logistic regression and decision tree modeling. This approach provides a more comprehensive understanding of the quantitative relationships between these factors and cognitive frailty, addressing the importance of individual factors and their interactions through systematic learning of attribute features and the visualization of results in tree diagrams (Shen et al., 2024). Furthermore, given the ongoing debate about the predictive accuracy of logistic regression and decision tree models (Nusinovici et al., 2020; Miyazaki et al., 2024), we conducted a comparative analysis of their efficiency using the AUC value.

Our findings revealed that both logistic regression and decision tree modeling identified sleep quality, number of chronic diseases, depression, educational level, nutrition, and exercise as significant influencing factors of cognitive frailty among older adults in nursing homes. Furthermore, logistic regression analysis indicated that age and intellectual activity were additional influencing factors. However, the decision tree analysis did not highlight the influence of these two factors. This discrepancy may be attributed to the logistic regression model’s ability to capture correlations between variables, while the decision tree model accounts for interactions and relationships among variables, providing a detailed functional form for each subcategory and offering a wealth of information (Zhang et al., 2022).

Regarding the comparative efficiency of logistic regression and decision tree modeling, the results demonstrated that both methods achieved AUC values exceeding 0.7, indicating strong classification performance for predicting the risk factors of cognitive frailty among older adults in nursing homes. Moreover, logistic regression exhibited high specificity, while decision tree analysis demonstrated high sensitivity. Therefore, the combined application of these two methods can enhance the analysis of factors influencing cognitive frailty in older adults residing in nursing homes.

#### Influence of sociodemographic factors on cognitive frailty among elderly people in nursing homes

In our study, the logistic regression model identified age as a risk factor for CF among older adults in nursing homes, while the decision tree model did not. This discrepancy may be attributed to the decision tree’s comprehensive analysis of interactions between different factors. Compared to other factors, the relative effect of age on cognitive frailty was relatively small and deemed less important, leading to its exclusion as an interfering factor in the analysis. Despite this, considering the existing research evidence, we suggest retaining age as a risk factor for cognitive frailty among older adults in nursing homes. For example, studies have demonstrated a strong association between age and cognitive decline (Furtado et al., 2020), with older individuals, particularly those over 80 years old, at greater risk of cognitive frailty (Zhang et al., 2024). Several investigations have explored the relationship between frailty, cognitive impairment, and cognitive frailty, highlighting the role of aging in their development (Godin et al., 2017). Aging can lead to the dysfunction of multiple systems, reducing the body’s physiological reserves and increasing vulnerability to frailty (Arai et al., 2018). Additionally, age-related hearing loss and hippocampal shrinkage contribute to a gradual decline in brain function, suggesting that brain aging may be a pathological mechanism underlying cognitive decline (Panza et al., 2018).

Consistent with the literature (Setiyani and Iskandar, 2022), the results of both logistic regression and decision tree analysis revealed a significant association between education level and cognitive function among nursing home residents. Older individuals with higher education levels often engage in activities such as reading books and newspapers, maintaining a relatively active state of their brain cells for extended periods. This enhanced brain activity can improve the brain’s compensatory abilities for pathological aging, thereby reducing the risk of cognitive dysfunction (Maharani et al., 2023). This finding further elucidates why a low education level is a risk factor for cognitive frailty in our study. Elderly individuals with higher education levels tend to possess a stronger sense of health and are more likely to proactively seek health-related information and engage in health-promoting behaviors, consequently decreasing the risk of cognitive frailty (Bakker et al., 2017). Moreover, the decision tree analysis in our study indicated that the prevalence of cognitive frailty was highest among older adults with insomnia and illiteracy, suggesting that these factors may increase the risk of cognitive frailty among nursing home residents.

#### Influence of life behavior habit factors on cognitive frailty among elderly people in nursing homes

The findings of this study, utilizing logistic regression and decision tree modeling, indicate that exercise is a significant factor influencing cognitive frailty among older adults in nursing homes. Various types of exercise have been shown to mitigate adverse outcomes in nursing home residents, including falls (Dyer et al., 2023), frailty (Sahin et al., 2022), and cognitive decline (Hirt et al., 2024). Given the high prevalence of physical inactivity among Chinese nursing home residents, reaching 88.46%, tailored interventions are essential to promote physical activity in this population (Shi et al., 2024). A previous study (Angulo et al., 2020) has demonstrated that exercise can regulate bone metabolism, enhance skeletal muscle contractile function, and delay age-related bone loss and muscle strength decline, thereby maintaining better physical function and reducing the incidence of frailty. Additionally, exercise can increase brain blood circulation through complex neural reflex pathways, reshaping brain function, delaying brain atrophy, and slowing cognitive decline (Angulo et al., 2020). The decision tree analysis further revealed that regular exercise can reduce the prevalence of cognitive frailty in individuals with insomnia and at least a secondary school education, while occasional or no exercise may increase the risk. These findings highlight the crucial role of regular exercise in mitigating cognitive frailty among older adults in nursing homes, particularly those with lower educational levels.

While logistic regression and decision tree models identified differing influences of intellectual activities on cognitive frailty in nursing homes, with decision tree analysis indicating a lack of association, previous research (Li et al., 2022a) has highlighted the positive impact of intellectual activities on cognitive function. Effective cognitive stimulation through intellectual activities can promote continuous brain activity, enhance brain cell function, and strengthen neural network connections, thereby delaying cognitive decline. A recent study suggested that intellectual activities may have varying moderating effects on the relationship between age and memory performance (Karsazi et al., 2024), emphasizing the importance of nursing institutions in encouraging older adults to actively participate in exercise and intellectual activities to prevent or slow down the onset of cognitive frailty.

#### Influence of physical health factors on cognitive frailty among elderly people in nursing homes

Sleep quality, number of chronic diseases, and nutrition emerged as significant factors influencing cognitive frailty among older adults in our study’s nursing homes. Notably, decision tree analysis revealed sleep quality as the most strongly correlated and influential factor. Previous research has highlighted the potential for long-term sleep disorders to induce cardiovascular, cerebrovascular, or degenerative neurological diseases, known risk factors for physical frailty. Furthermore, sleep disorders can impair cognitive function through mechanisms such as inflammation and vascular lesions (Nakakubo et al., 2018; Hu et al., 2021), suggesting that improving sleep quality could be a valuable target for mitigating cognitive frailty in nursing home residents. A study (Liu et al., 2022) demonstrated a significant association between poor sleep quality and long nap duration with a higher risk of cognitive frailty, suggesting that reducing nap time might be beneficial in reducing the incidence of cognitive frailty. The prevalence of chronic diseases is often elevated among older adults, with studies indicating that 55–98% of elderly individuals suffer from multimorbidity (Anderssen-Nordahl et al., 2024). Our findings revealed a higher prevalence of cognitive frailty among older adults with more chronic diseases. Chronic diseases can exacerbate the decline of various organ functions in the elderly, increasing their susceptibility to physical frailty (Zazzara et al., 2019). Additionally, common chronic diseases like hypertension and diabetes can synergistically damage vascular endothelial cells, leading to brain hypoxia and oxidative damage, ultimately contributing to cognitive decline (Chu et al., 2022). According to a recent survey (Liu et al., 2020), a significant proportion of nursing home residents in China (5.1 and 55.6%, respectively) were malnourished or at risk of malnutrition, highlighting the prevalent poor nutritional status among this population. Multiple studies have demonstrated that inadequate nutrition predisposes individuals to cognitive frailty (Gómez-Gómez and Zapico, 2019). Malnutrition can lead to weight loss and muscle tone decline, both important physical frailty indicators (Lorenzo-López et al., 2017). Moreover, deficiencies in specific nutrients, such as vitamins and micronutrients, can contribute to cognitive decline (Scarmeas et al., 2018).

#### Influence of social psychological factors on cognitive frailty among elderly people in nursing homes

Consistent with the literature (Zou et al., 2023), depression emerged as a factor influencing cognitive frailty among older adults in nursing homes. A recent study (Ruan et al., 2020) suggested that depression can elevate levels of chronic inflammatory factors in the body. These inflammatory factors not only directly affect the musculoskeletal system but can also cross the blood–brain barrier, leading to an increase in amyloid protein in the brain and subsequent impairment of cognitive function. Furthermore, the decision tree analysis in this study revealed an interaction between suspicious insomnia and depression. In individuals with suspicious insomnia, depression was associated with a higher prevalence of cognitive frailty.

This study employed logistic regression and decision tree modeling to investigate the influential factors of cognitive frailty among older Chinese adults residing in nursing homes. The findings may offer novel insights into the prevention of cognitive frailty in this population. However, certain limitations should be acknowledged. Firstly, the cross-sectional study design precludes the establishment of a causal relationship between cognitive frailty and the identified risk factors. Secondly, the relatively limited scope of the investigation may restrict the generalizability of the research conclusions to other regions. Thirdly, the reliance on self-reported data and the exclusion of participants with missing values could introduce information and selection biases. Future research could address these limitations by expanding the sample size, conducting multi-center studies, and incorporating paraclinical investigations such as blood work and brain imaging. These measures would enhance the generalizability and clinical significance of the findings.

## Conclusion

Overall, our study identified age, education level, exercise, intellectual activity, sleep quality, number of chronic diseases, nutrition, and depression as factors influencing cognitive frailty among older adults in nursing homes. Among these, sleep quality emerged as the most significant factor. Based on these findings, we propose several recommendations: first, nursing institutions should prioritize the assessment and early screening of cognitive frailty, particularly among older adults with low education levels. Second, nursing institutions should actively promote exercise and intellectual activities, encouraging older adults to participate regularly to prevent or delay cognitive decline. Third, healthcare professionals in nursing homes should address sleep and nutrition issues, creating a conducive sleep environment and implementing scientifically sound dietary nutrition programs to improve the sleep and nutritional status of older adults. For individuals with multiple chronic diseases, collaboration with relevant medical institutions to develop tailored treatment, nursing, and rehabilitation programs can enhance health management abilities and potentially delay or improve cognitive decline. Finally, greater attention should be paid to psychological evaluation and targeted health education for older adults with poor sleep quality to prevent or delay the onset of cognitive decline.

The results of the logistic regression and decision tree models were consistent, efficient, and demonstrated distinct advantages. Logistic regression analysis, after controlling for confounding factors, explored the linear relationship between independent and dependent variables. The output odds ratio (OR) values provided quantitative insights into the dependency between independent variables and the risk of cognitive frailty (Chen et al., 2022), offering a clearer understanding of the influence of various factors. In contrast, decision tree analysis generated a tree graph that directly visualized the importance of each risk factor in predicting cognitive frailty. Both methods effectively identified risk factors for cognitive frailty among older adults in nursing homes, as evidenced by AUC values greater than 0.7. The combined application of logistic regression and decision tree models can leverage their complementary strengths, providing a more comprehensive understanding of the factors influencing cognitive frailty in older adults in nursing homes. Healthcare professionals should actively monitor these factors, identify high-risk groups early, and implement targeted and holistic preventive interventions to prevent or delay the onset of cognitive decline, thereby improving the quality of life for older adults in their later years.

## Data Availability

The original contributions presented in the study are included in the article/supplementary material, further inquiries can be directed to the corresponding authors.
